# Type B Aortic Dissection Following Abdominal Aortic Aneurysm Repair in Loeys–Dietz Syndrome: A Novel *TGFBR1* Variant

**DOI:** 10.1155/humu/2951304

**Published:** 2026-05-18

**Authors:** Yuchong Zhang, Shouji Qiu, Chengkai Hu, Li Zhang, Wei Guo, Weiguo Fu, Lixin Wang

**Affiliations:** ^1^ Department of Vascular Surgery, Zhongshan Hospital, Fudan University, Shanghai, China, fudan.edu.cn; ^2^ Vascular Surgery Institute of Fudan University, Shanghai, China, fudan.edu.cn; ^3^ National Clinical Research Center for Interventional Medicine, Zhongshan Hospital, Fudan University, Shanghai, China, fudan.edu.cn; ^4^ Department of Laboratory Medicine, Zhongshan Hospital, Fudan University, Shanghai, China, fudan.edu.cn

**Keywords:** aortic dissection, genotype–phenotype correlation, Loeys–Dietz syndrome, novel variant, *TGFBR1*

## Abstract

Loeys–Dietz syndrome (LDS) is an autosomal dominant connective tissue disorder characterized by aggressive aortic pathology, primarily caused by pathogenic variants in genes such as *TGFBR1*. We report a 44‐year‐old female with a known LDS diagnosis who presented with a symptomatic, rapidly expanding abdominal aortic aneurysm (44.8 mm) with concurrent iliac and renal artery involvement. Given the high rupture risk, she underwent open abdominal aortic reconstruction. However, she subsequently developed a Type B aortic dissection nearly 2 months after the initial repair, necessitating a second open thoracic surgery. Whole‐exome sequencing confirmed a novel, de novo heterozygous missense variant in the *TGFBR1* gene: c.1051G>T (p.Asp351Tyr), located within the highly conserved kinase domain and classified as likely pathogenic. Structural modeling suggested that this variant enhances binding affinity to SMAD2, and immunohistochemistry of the patient′s aortic tissue confirmed hyperactivation of the TGF‐*β* pathway via increased SMAD2/SMAD3 phosphorylation. This case expands the pathogenic variant spectrum of *TGFBR1*‐related LDS. Furthermore, it provides valuable clinical insights into the management of abdominal aortic aneurysms in LDS, suggesting that symptoms and rapid growth may warrant surgical intervention before conventional diameter thresholds are met. It also serves as a stark reminder of the residual vascular fragility, reinforcing the need for lifelong, whole‐body vascular surveillance.

## 1. Introduction

Aortic diseases represent a heterogeneous group of disorders with multifactorial etiologies, encompassing genetic, immunological, and metabolic factors, among which genetic predisposition is a crucial determinant. Loeys–Dietz syndrome (LDS), first described by Loeys et al. in 2005 [1], is a rare autosomal dominant connective tissue disorder, initially classified as a variant of Marfan syndrome (MFS) due to overlapping phenotypic features. However, LDS is now recognized as a distinct clinical entity (OMIM #609192). It is characterized by arterial tortuosity, aortic aneurysms and dissections, distinctive skeletal abnormalities (such as scoliosis and talipes equinovarus), and craniofacial features (e.g., bifid uvula and craniosynostosis) [2]. Importantly, LDS is associated with more aggressive aortic pathology than MFS, with a significantly higher propensity for widespread arterial aneurysms and dissections extending beyond the aortic root into the abdominal and peripheral vasculature [3–5].

Germline pathogenic variants in the transforming growth factor beta receptor 1 (*TGFBR1*) or transforming growth factor beta receptor 2 (*TGFBR2*) genes are the primary molecular causes of LDS, accounting for over 75% of cases [2]. *TGFBR1* encodes a serine/threonine kinase receptor that forms a heteromeric complex with *TGFBR2*, initiating the transforming growth factor‐*β* (TGF‐*β*) signaling cascade [6]. Ligand binding to *TGFBR2* induces phosphorylation of *TGFBR1*, activating its kinase domain and facilitating the phosphorylation of SMAD2/SMAD3 transcription factors to regulate genes critical for cellular growth, differentiation, and vascular homeostasis. Despite the common phenotypic features of LDS, significant clinical variability exists, which correlates with the specific nature and location of *TGFBR1/TGFBR2* variants, suggesting a robust genotype–phenotype relationship [7].

Although prophylactic surgical thresholds for aortic root aneurysms in LDS are well‐established, the management of abdominal aortic aneurysms (AAAs) remains a significant clinical gray area. Current guidelines lack definitive diameter thresholds for AAA intervention in patients with heritable aortic diseases (HADs), often relying on individualized factors such as a rapid growth rate and symptomatic presentation [8]. Furthermore, the natural history of the residual aorta following major surgical reconstruction in these patients is fraught with the risk of subsequent catastrophic events, highlighting the extreme fragility of the entire vascular tree.

In this study, we present a longitudinally followed Chinese female patient with a known diagnosis of LDS who presented with concurrent aneurysms involving the abdominal aorta, iliac arteries, and renal arteries. Driven by rapid aneurysmal expansion and symptomatic presentation, she underwent open abdominal aneurysm repair, only to develop an acute Type B aortic dissection (TBAD) shortly thereafter. Whole exome sequencing (WES) confirmed a novel, de novo *TGFBR1* variant (c.1051G>T, p.Asp351Tyr) within the highly conserved kinase domain. We hypothesize that this variant disrupts kinase domain activity, overactivating TGF‐*β* signaling and contributing to the complex and highly aggressive aortic phenotype observed in this patient.

## 2. Materials and Methods

### 2.1. Study Subject and Ethical Statement

The study was conducted in accordance with the Declaration of Helsinki and was approved by the institutional review board of Zhongshan Hospital, Fudan University (B2019‐231R). The proband, a 44‐year‐old female, provided written informed consent for the collection of clinical data, genetic analysis, and publication of the research findings. Control aortic tissue samples were obtained from heart transplant recipients without known connective tissue disorders, with prior informed consent and institutional ethical approval.

### 2.2. WES and Sanger Sequencing

Peripheral venous blood (3 mL) was collected from the proband and her parents into EDTA‐K2 anticoagulant tubes. Genomic DNA was extracted using a commercial DNA extraction kit (TIANGEN Biotech, Beijing, China) following the manufacturer′s instructions. The qualified genomic DNA was fragmented to construct a whole‐exome library. Target enrichment of exonic regions was performed, and the resulting library was sequenced on an MGISEQ‐2000 platform. To validate the WES findings and determine the variant′s origin, the candidate missense variant in *TGFBR1* was analyzed in the proband and her parents by conventional Sanger sequencing using specifically designed primers flanking the target region.

### 2.3. Protein Structure Modeling and Molecular Docking

The three‐dimensional structures of the wild type (WT) and p.Asp351Tyr mutant *TGFBR1* kinase domains in complex with SMAD2 and SMAD3 were predicted using AlphaFold 3 [9]. The resulting models were visualized and analyzed using PyMOL to investigate changes in protein–protein interactions, particularly the formation and alterations of hydrogen bonds at the *TGFBR1*–SMAD2/3 interface.

### 2.4. Histology, Immunohistochemistry (IHC), and Immunofluorescence (IF)

Aortic tissue specimens from the patient and healthy controls were fixed in 4% paraformaldehyde (PFA), paraffin‐embedded, and sectioned at 4 *μ*m thickness. For histological assessment, sections were stained with hematoxylin and eosin, Verhoeff–Van Gieson, and Masson′s trichrome. For IHC, sections underwent deparaffinization, rehydration, and heat‐induced antigen retrieval. After blocking, they were incubated overnight at 4°C with a primary antibody against phosphorylated SMAD2/SMAD3 (ABclonal, AP0548). Signals were detected using an HRP‐conjugated secondary antibody and a DAB substrate kit, followed by counterstaining with hematoxylin. For IF, sections were processed similarly for antigen retrieval and blocking. They were then incubated with primary antibodies against p‐SMAD2/SMAD3 (ABclonal, AP0548) and *ACTA2* (Servicebio, GB13044‐50). After washing, sections were incubated for 1 h at room temperature with a fluorescently labeled secondary antibody. Slides were mounted with an antifade mounting medium. Images were captured using an Olympus BX53 confocal microscope.

## 3. Results and Discussion

### 3.1. Clinical Description

The patient was a 44‐year‐old Chinese female who presented with a complex systemic phenotype highly characteristic of heritable connective tissue disorders. Her physical examination revealed a classic marfanoid habitus, including slender fingers with a positive thumb sign (arachnodactyly), severe lumbar scoliosis, flat feet (pes planus), and marked joint hypermobility. Craniofacial assessment noted distinct facial asymmetry. Dermatological examination showed velvety, thin, and translucent skin with easily visible underlying veins, accompanied by a history of easy bruising. Her ophthalmologic history included myopia and a noticeable increase in eye floaters. She also reported experiencing frequent palpitations. Abdominal imaging incidentally revealed multiple cysts in the liver, spleen, and pancreas. She had no family history of aortic aneurysm, dissection, or sudden death. Notably, the patient retrospectively reported a visible, pulsatile abdominal mass shortly after a full‐term pregnancy in 2004 (at age 24), though she did not seek medical evaluation at that time.

In July 2018, she experienced sudden right scapular pain and sought medical attention at a local hospital. Imaging revealed an AAA measuring 33.5 mm in maximum diameter, while her iliac and renal arteries were completely normal at that time. Suspecting a heritable connective tissue disorder such as MFS, the patient and her family independently pursued genetic testing through a third‐party commercial laboratory, which identified a pathogenic variant in the TGF‐*β* signaling pathway. Crucially, cascade screening of her parents, sister, and daughter yielded negative results, confirming the de novo origin of the mutation. Despite the genetic diagnosis, prophylactic medications (such as beta‐blockers or angiotensin receptor blockers) were not initiated at that time because her blood pressure (105/67 mmHg) and heart rate were strictly within normal limits.

The patient was managed conservatively with regular imaging surveillance. The AAA grew slowly to 38 mm by 2021 and remained stable at 39 mm in July 2023. However, in October 2024, she presented to our center with recurrent right scapular pain. A repeat CTA demonstrated a rapid expansion of the AAA, reaching a maximum diameter of 44.8 mm and a length of 121 mm, accompanied by newly developed concurrent dilatation of the bilateral common iliac arteries (11.6 and 11.4 mm) and multiple renal artery aneurysms. To definitively confirm the molecular etiology before undertaking complex open surgery, WES was repeated at our institution, which formally verified the de novo *TGFBR1* variant and established the definitive diagnosis of LDS.

Although the absolute diameter of the AAA (44.8 mm) was well below the conventional 50–55‐mm threshold for sporadic aneurysms, a proactive multidisciplinary decision was made to proceed with elective open surgery. This individualized approach was driven by three high‐risk features: the patient′s symptomatic presentation (recurrent right scapular pain), rapid aneurysmal growth (nearly 7 mm expansion within 15 months), and her confirmed LDS diagnosis. In January 2025, she underwent an open AAA resection with prosthetic vascular graft replacement and abdominal aorta–renal artery bypass grafting. Open surgery was selected over endovascular repair due to the complex anatomy and the proven durability of open reconstruction in patients with connective tissue disorders.

**Figure 1:**
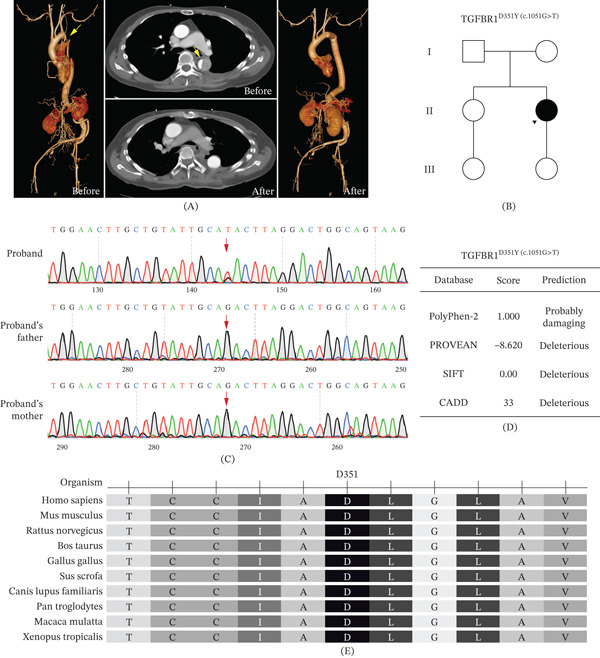
Clinical and genetic findings in the patient with Loeys–Dietz syndrome. (A) Computed tomography angiography (CTA) images of the patient. Three‐dimensional reconstructions (before and after) demonstrate extensive aneurysmal dilatation of the abdominal aorta and iliac arteries. Axial views (center) show a Type B aortic dissection in the descending thoracic aorta with a clear intimal flap (yellow arrows). (B) Pedigree of the family showing three generations. The proband (II‐2) is the sole affected individual, consistent with a de novo pathogenic variant. Squares represent males; circles represent females; filled symbol indicates an affected individual. (C) Sanger sequencing chromatograms confirming the heterozygous c.1051G>T variant in the proband and its absence in her unaffected parents, establishing its de novo origin. The red arrow indicates the position of the variant. (D) In silico prediction of the pathogenicity for the *TGFBR1* p.Asp351Tyr variant. Multiple computational tools (PolyPhen‐2, PROVEAN, SIFT, and CADD) consistently predict a deleterious effect on protein function. (E) Multiple sequence alignment of the TGFBR1 protein shows that the aspartic acid residue at position 351 (D351, highlighted) is highly conserved across different vertebrate species, indicating its functional importance.

The patient recovered well initially. However, in late February 2025, following an episode of emotional stress, she experienced sudden, severe chest pain described as a “tightening” sensation. Emergency CTA revealed an acute TBAD without organ malperfusion. Given the acute and uncomplicated nature of the dissection, the surgical team opted for strict medical management in the intensive care unit for 3 weeks. This deliberate delay was intended to control her hemodynamics, allow the acute inflammatory edema of the fragile aortic wall to subside, and optimize her physical condition following the recent major abdominal surgery. Once stabilized, she underwent a second major operation in March 2025: an open thoracic aortic partial resection with prosthetic graft replacement and diaphragmatic plication. During this procedure, a previously undiagnosed patent ductus arteriosus (PDA), which had been missed on initial screening echocardiograms, was identified and surgically ligated. The patient was discharged in April 2025 and is currently maintained on oral bisoprolol (5 mg daily) with close lifelong imaging surveillance. **(**Figure 1A**)**.

### 3.2. WES and Conservation Analysis

WES was performed using the aforementioned protocol to definitively confirm the genetic etiology. Molecular genetic screening revealed a novel heterozygous missense mutation in Exon 6 of *TGFBR1* (NM_006412.4: c.1051G>T; p.Asp351Tyr), located within the evolutionarily conserved protein kinase domain (Figures 1B, 1C, and 1E). Sanger sequencing of the proband and her unaffected parents confirmed the absence of this variant in the parents, establishing its de novo origin (Figure 1C).

In silico analyses predicted this variant to be “probably damaging” by PolyPhen‐2 and “deleterious” by PROVEAN, SIFT, and CADD algorithms (Figure 1D) [10, 11]. According to ACMG guidelines [12], the c.1051G>T variant was classified as pathogenic (classification criteria: PP3‐moderate, PM2‐supporting, PM5‐supporting, and PS2). This variant has not been reported in the 1000 Genomes Project, gnomAD, Exome Sequencing Project (ESP) databases, or publications, and has not been previously associated with AAA pathogenesis in existing literature.

### 3.3. Protein Structure Modeling and Docking

**Figure 2:**
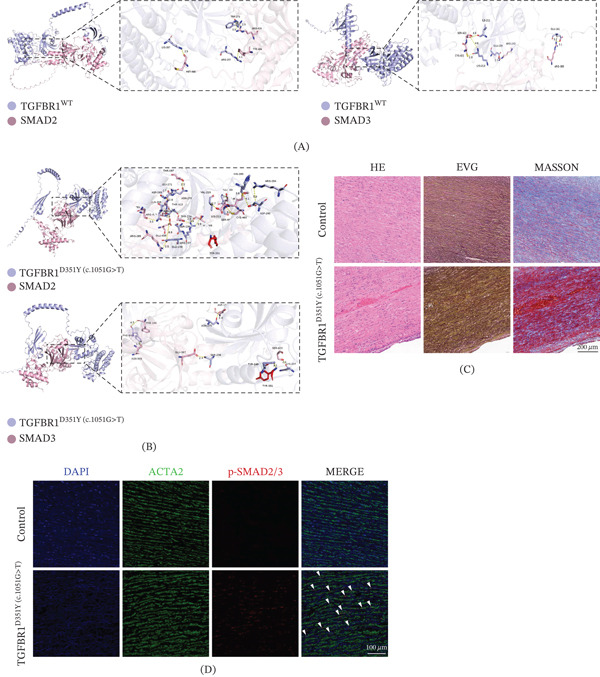
Molecular modeling and pathological consequences of the *TGFBR1* p.Asp351Tyr variant. (A) Predicted structural models of the interaction between wild‐type (WT) *TGFBR1* and its substrates SMAD2 (left) and SMAD3 (right). Insets provide detailed views of the key interacting residues at the binding interface. (B) Predicted structural models of the p.Asp351Tyr (D351Y) mutant *TGFBR1* with SMAD2 (top) and SMAD3 (bottom). The mutation is predicted to significantly alter the binding interface and increase interactions with SMAD2, whereas the interaction with SMAD3 remains largely unchanged. (C) Histopathological analysis of the patient′s aortic wall compared to a control aorta. Hematoxylin and eosin (HE) staining reveals disorganization of the medial layer. Elastica van Gieson (EvG) staining shows severe fragmentation and loss of elastic fibers. Masson′s trichrome staining indicates increased collagen deposition (blue). Scale bar = 200 *μ*m. (D) Immunofluorescence staining for phosphorylated SMAD2/SMAD3 (p‐SMAD2/SMAD3, red), a marker of TGF‐*β* pathway activation. Compared to the control, the patient′s aortic tissue shows a marked increase in nuclear p‐SMAD2/SMAD3 signal (indicated by white arrowheads) in smooth muscle cells (*ACTA2*, green). Nuclei are counterstained with DAPI (blue). Scale bar = 100 *μ*m.

To further explore the potential structural mechanisms underlying the pathogenicity of c.1051G>T, we performed exploratory structural predictions of human *TGFBR1* (WT and p.Asp351Tyr variant) in complex with SMAD2 and SMAD3 and conducted molecular docking simulations based on AlphaFold 3 [9]. The interface between *TGFBR1* and SMAD2/SMAD3 primarily consists of noncovalent protein–protein interactions, with hydrogen bonds being the predominant interaction type. WT *TGFBR1* residues involved in hydrogen bonding, such as Ile211, Lys213, and Arg273, are all located within the protein kinase domain (Figure 2A). These findings suggest that WT *TGFBR1* predominantly interacts with SMAD2 and SMAD3 intracellularly via its protein kinase domain.

Subsequently, we analyzed the predicted effect of the p.Asp351Tyr mutation on the interaction between *TGFBR1* and SMAD2/SMAD3 (Figure 2B). Compared to the WT *TGFBR1*, the interaction pattern with SMAD3 remained largely unchanged in the p.Asp351Tyr mutant. The mutant retained most of the key residue interactions with SMAD3, indicating that the p.Asp351Tyr mutation likely has a minimal effect on the binding affinity between *TGFBR1* and SMAD3.

However, the simulated interaction between the p.Asp351Tyr mutant and SMAD2 underwent significant changes. We observed that the mutant is predicted to form new hydrogen bond interactions with SMAD2, primarily involving multiple residues within the protein kinase domain of the p.Asp351Tyr mutant. Although these in silico findings are derived from a single case, they provide a theoretical framework suggesting that the mutation may lead to an increased binding affinity for SMAD2, potentially altering downstream signal transduction.

### 3.4. Histological Analysis and IF Staining

Compared with control specimens obtained from heart transplant recipients, the aortic wall thickness at the patient′s dissection site was increased. Histological evaluation revealed severe medial disorganization, elastic fiber degeneration, and collagen deposition, along with an increased number of vessels in the adventitia (Figure 2C). In addition, IF staining demonstrated that the levels of phosphorylated SMAD2/SMAD3 in the aortic media/adventitia were significantly upregulated compared with the control group (Figure 2D). These localized histological findings are consistent with our structural predictions, suggesting an aberrant hyperactivation of the canonical TGF‐*β* signaling pathway in the diseased aortic tissue of this specific patient.

## 4. Discussion

In this study, we report a novel, de novo missense variant, c.1051G>T (p.Asp351Tyr), in *TGFBR1*, identified in a young female patient diagnosed with LDS. While the genetic discovery expands the mutational spectrum of LDS, the longitudinal clinical trajectory of this patient provides critical teaching opportunities regarding the medical and surgical management of HADs. This complex trajectory spans from an initial delayed diagnosis to a symptomatic AAA and a subsequent postoperative TBAD.

*TGFBR1* and *TGFBR2* pathogenic variants are the molecular hallmarks of LDS, classified as Types I and II, respectively. Subsequently, variants in other genes, including SMAD3 (Type III), *TGFB2* (Type IV), SMAD2, and *TGFB3* (Type V), have also been linked to LDS. Our patient′s *TGFBR1* c.1051G>T variant corresponds to LDS Type I and aligns with the typical phenotype of aortic aneurysms/dissections and scoliosis [2]. However, unlike MFS, which predominantly affects the aortic root, LDS is characterized by a significantly higher penetrance of widespread arterial events, including abdominal and peripheral aneurysms. A recent large‐cohort study by Calderon‐Martinez et al. highlighted that patients with pathogenic variants in the TGF‐*β* pathway face an exceptionally high and early risk of extra‐aortic arterial events, underscoring the systemic nature of the vascular fragility [4].

Furthermore, a 2025 study by Abdul Nabi et al. identified significant sex‐based differences in LDS, reporting that males are generally more susceptible to severe macrovascular complications such as AAAs and dissections than females [13]. The highly aggressive and complex vascular trajectory observed in our female patient underscores that specific *TGFBR1* variants can precipitate malignant phenotypes irrespective of epidemiological sex trends, necessitating vigilant monitoring for all patients.

Most pathogenic *TGFBR1* variants are missense mutations that cluster within the kinase domain, impairing canonical TGF‐*β* signaling [14, 15]. As recently demonstrated by Stathori et al., in silico structural modeling serves as a powerful and increasingly recognized tool to elucidate genotype–phenotype correlations in LDS by revealing how specific missense variants disrupt protein 3D folding and stability [16].

In our study, exploratory molecular docking analyses revealed that the p.Asp351Tyr variant lies within this domain and is predicted to enhance binding to the MH2 domain of SMAD2. Histopathological and IF examinations of the patient′s aortic tissue corroborated these in silico findings, showing structural medial disorganization and elevated levels of phosphorylated SMAD2/SMAD3. However, the exact mechanisms by which altered TGF‐*β* signaling contributes to aneurysm formation remain highly controversial, with evidence suggesting both protective and pathogenic roles depending on the disease stage [17–19]. We acknowledge that findings from a single case cannot definitively resolve this complex mechanistic debate; rather, they provide a localized snapshot of pathway hyperactivation in the diseased aorta.

From a clinical perspective, the longitudinal trajectory of this case highlights the critical evolution of clinical management standards for LDS and provides crucial teaching points.

First, the rapid multiterritory progression of the disease underscores the absolute necessity of continuous whole‐body surveillance. Although the patient′s iliac and renal arteries were normal during the initial imaging in 2018, a repeat head‐to‐pelvis CTA in 2024 revealed newly developed, extensive bilateral iliac and renal artery aneurysms. This aggressive systemic evolution powerfully reinforces the 2022 ACC/AHA and 2024 ESC/ESVS guidelines, which mandate not only baseline screening but strict, lifelong periodic imaging of the entire arterial tree in LDS patients [5, 20, 21]. Furthermore, the patient′s PDA was missed during initial screening echocardiograms and only identified incidentally during thoracic surgery. Given the high prevalence of congenital cardiac anomalies in LDS, this highlights the need for advanced cardiovascular imaging when standard echocardiography is suboptimal.

Second, prophylactic hemodynamic‐lowering therapy was withheld in 2018 because the patient was normotensive. Although withholding medication in normotensive patients might have reflected historical practices in nonspecialized settings, current standards dictate otherwise. It is now imperative that medical therapy (e.g., beta‐blockers or ARBs) be initiated immediately upon genetic confirmation of LDS, regardless of baseline blood pressure, to mitigate long‐term catastrophic vascular fragility.

Third, the occurrence of an acute TBAD just 2 months after the major abdominal reconstruction underscores the extreme fragility of the residual aorta in LDS. The hemodynamic shifts and systemic stress induced by the initial open surgery likely acted as triggers for the subsequent dissection [22]. The multidisciplinary decision to observe the uncomplicated TBAD for 3 weeks prior to the second open surgery was a calculated strategy. It allowed the acute inflammatory edema of the aortic wall to subside, reducing tissue friability while optimizing the patient′s physical condition to endure a second major open thoracic repair.

This study has some limitations. Clinically, it is a retrospective report of a single case, and the surgical thresholds applied here require validation in larger, multicenter registries. Mechanistically, we rely primarily on computational and pathological methods to evaluate the impact of this mutation. Directly evaluating the effect of this mutation on TGF‐*β* signaling activity through in vitro molecular biology experiments would be more instructive.

## 5. Conclusions

We report a novel pathogenic variant, c.1051G>T (p.Asp351Tyr), in the *TGFBR1* gene in a Chinese female patient with LDS. Beyond expanding the genotypic spectrum, this case provides vital clinical insights. It reinforces the necessity of early prophylactic medication and lifelong whole‐body surveillance, illustrates that rapid growth and symptoms justify AAA surgical intervention before conventional diameter thresholds are met, and serves as a stark reminder of the lifelong risk of dissection in the residual aorta of patients with LDS.

## Author Contributions

Y.Z. designed the study, collected clinical data, performed experiments, analyzed the data, and drafted the manuscript. S.Q. contributed to data collection and analysis. C.H. contributed to data collection and performed the molecular modeling analysis. L.Z. and W.G. performed the genetic sequencing. W.F. and L.W. supervised the project, provided critical revisions to the manuscript, and were responsible for patient management. Y.Z. and S.Q. contributed equally to this work.

## Funding

This work was supported by the National Natural Science Foundation of China (Grant numbers 82270415 and U24A20651).

## Disclosure

All authors have read and approved the final version of the manuscript.

## Conflicts of Interest

The authors declare no conflicts of interest.

## Data Availability

Research data are not shared.
